# Chaos-Based Simultaneous Compression and Encryption for Hadoop

**DOI:** 10.1371/journal.pone.0168207

**Published:** 2017-01-10

**Authors:** Muhammad Usama, Nordin Zakaria

**Affiliations:** HPCC Service Center, Department of Computer & Information Sciences, Universiti Teknologi PETRONAS, Seri Iskandar, Tronoh, Perak, Malaysia; West Virginia University, UNITED STATES

## Abstract

Data compression and encryption are key components of commonly deployed platforms such as Hadoop. Numerous data compression and encryption tools are presently available on such platforms and the tools are characteristically applied in sequence, i.e., compression followed by encryption or encryption followed by compression. This paper focuses on the open-source Hadoop framework and proposes a data storage method that efficiently couples data compression with encryption. A simultaneous compression and encryption scheme is introduced that addresses an important implementation issue of source coding based on Tent Map and Piece-wise Linear Chaotic Map (PWLM), which is the infinite precision of real numbers that result from their long products. The approach proposed here solves the implementation issue by removing fractional components that are generated by the long products of real numbers. Moreover, it incorporates a stealth key that performs a cyclic shift in PWLM without compromising compression capabilities. In addition, the proposed approach implements a masking pseudorandom keystream that enhances encryption quality. The proposed algorithm demonstrated a congruent fit within the Hadoop framework, providing robust encryption security and compression.

## 1 Introduction

The term ‘data’ refers to symbols that are stored or transmitted. Some data symbols refer to a sequence, a segment, or a block. An application inserts or places data within a communication channel or in storage on a dedicated device. Data stored on storage devices or transferred over communication channels between computers or networks contains significant redundancy. Data compression reduces this redundancy to save physical disk space and minimize network transmission time. Decompression retrieves original data from compressed data and can be accomplished without data loss. Thus, data compression improves available network bandwidth and storage capacity, especially when the original data contains significant redundancy. Data compression is an important playground for information theory, relying on its core on entropy measurement and sequences complexity measurement [1] for optimal performance. Further, compression techniques require a pattern library and only work when both sender and receiver understand the library that is utilized. However, in general, compression does not employ secret key or password restrictions during compression and decompression, a deficiency that reduces the overall level of security. Its primary purpose is to lessen data redundancy. Hence, compressed data is vulnerable to unauthorized access and illegal use.

**Fig 1:**
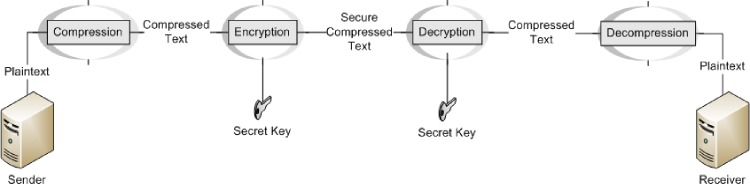
Legacy compression first approach.

**Fig 2:**
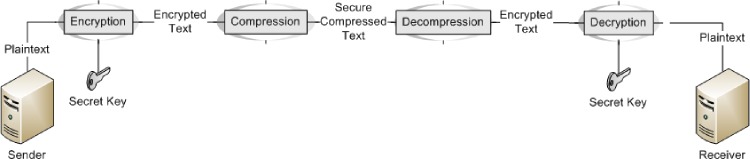
Legacy encryption first approach.

Data encryption is needed to achieve data security and keep the data unreadable and unaltered. Encryption techniques usually manipulate data as a function of randomly generated bits, bytes or blocks through a secret key. Naturally, the operational sequence can be one of two forms; it either compresses the data before encryption or encrypts the data before compression (See: Figs 1 and 2). The first form is more practically useful since the prime concern of data compression is to remove redundant data, an operation that also improves data security against statistical attack. The second form applies encryption first, which implies the generation of significant randomness in data with very little redundancy; thus deeming the latter compression step ineffective. Although sequential implementation of compression and encryption actually reduces data storage while improving data transmission bandwidth and security, it presently requires the output of one operation to be piped to the other, a potentially expensive operation. Hence, there is growing interest in the investigation of combined algorithm for data compression and encryption. In this paper, a tightly integrated compression and encryption scheme is presented in Hadoop that incorporates contemporary offerings from chaos theory as well as cryptography and compression studies.

This paper is organized as follows: Section II presents related literature, Section III describes the proposed algorithm in detail and Section IV presents experimental analysis and validation results. Finally, Section V concludes the work.

## 2 Related Works

The contemporary ‘Big Data’ trend signifies an exponential and global accommodation of previously unimaginable volumes of data that are instantaneously made available at low cost. Big Data can be structured or unstructured and generally exceeds the processing capabilities of stand-alone systems. User and market demands promise to ensure the unabated continuance of this trend with greater expectations and even more diverse application domains [2–12]. Platform-wise, the Hadoop framework has been playing a leading role in supporting Big Data needs. As an open source Apache Project, it implements a MR (MapReduce) processing paradigm together with the Hadoop Distributed File System (HDFS) [13] [14], a combination that enables massive data processing by thousands of hardware commodities.

The Hadoop framework is designed to ensure highly fault-tolerant and scalable access to application data. Datasets are distributed over a large commodity cluster [15] in small blocks for both storage and processing. These data blocks are replicated for fault tolerance. Security, however, has been lacking and solely dependent on operating systems’ access controls and permission protocols [15]. Such security measures are grossly inadequate when challenged by sophisticated, targeted attacks and insider threats. Hadoop is commonly deployed in public cloud services such as Yahoo, Amazon, and Google. Given the popularity of such services and the tremendous volumes of confidential data stored, it is critically necessary for Hadoop to provide sufficient security guarantees for both data storage and processing.

Another significant challenge is that of network bottlenecks caused by large volumes of I/O data processing. MR jobs for such large datasets require considerable time due to massive numbers of I/O operations during data transfers between cluster nodes. Enabling data compression throughout the MR process pipeline can be useful in reducing I/O operations costs and consumption of network resources [16]. Hence, both data compression and encryption are critical in Hadoop. However, compression and encryption as separate processes should be avoided to prevent significant data volumes being piped between both operations. An algorithm that seamlessly combines data compression and encryption is therefore worth pursuing, especially in the context of the Hadoop platform, which is the main contribution of this paper.

### 2.1 Encryption

Basic security features are already available for the Hadoop framework. These include file and directory permissions along with required user authentication and group resolution when accessing a Hadoop cluster [17]. However, user authentication and group access controls still do not guarantee a vigorous level of security. Users can still access each other’s resources during Hadoop execution. Thus, more sophisticated security measures based on data encryption are essential but relatively few works have focused on data encryption in Hadoop [17–21]. A security design proposed by [17] for Hadoop provided for strong mutual authentication and access controls using Kerberos. This approach buffered data in encrypted clusters before sending them to HDFS for a write operation. Another group [18] proposed an application-level encryption service for MR to support a file system that assumed pre-uploaded plaintext in HDFS. Security architecture for a private storage cloud based on HDFS was proposed by [20] to encrypt sensitive data for accommodation within a private storage cluster based on security protocols from the Trusted Platform Module (TPM). In [22], a differential privacy protection scheme was presented that removed the ability of deleting data by an attacker. Nevertheless, these techniques cannot be considered ‘protected’ because the data is stored in clear text format within a centralized server.

A bi-hybrid data encryption scheme for Hadoop (i.e., HDFS-RSA with HDFS-Pairing) was proposed by [19]. However, both components required considerable reading and writing overhead, far more than generic HDFS, especially during writing operations for encrypted HDFS where performance cost doubled compared to generic HDFS writing ops. A secure Hadoop with encrypted HDFS was proposed by [21] that added standard AES encryption and decryption modules as ‘built-in’ HDFS classes. This approach secured MR jobs in HDFS but increased performance overhead by 7% compared to generic HDFS–I/O ops. Overall, native encryption modules for the Hadoop framework have not been fully employed or tested [21]. It has also been reported that future Hadoop software releases will include encryption [23].

### 2.2 Compression

Since data compression techniques eliminate redundancy and data duplication [24], encrypted or random data cannot be compressed further. Compression techniques involve three processing stages: 1) data preprocessing, 2) removal of data duplication, and 3) bits’ reduction. Hadoop supports multiple compression formats for all I/O data and compression is applied when the MR reads or writes data, which has some advantages. It reduces the I/O operation load, which is important since MR jobs are almost always I/O bound. Furthermore, it improves cluster utilization via space reduction and speeds data transfers across the network.

However, these benefits hold data compression and decompression CPU overhead costs. The CPU usage increases when MR jobs run compressed data [25] because decompression is required before Mapper and Reducer processing. Hence, tradeoffs are required between storage savings, faster I/O, and enhanced network bandwidth increases in CPU loads [26]. Nonetheless, enabling data compression in multiple phases of an MR Job has been shown to increase overall Hadoop application performance by ~65% [16]. In Hadoop, a codec—a compiled Java class invoked by MR classes—is used for compression and decompression. Hadoop also supports many compression codecs, each with different characteristics and methods of eliminating data redundancy and duplication. Hadoop’s standard built-in codecs include Deflate, Gzip, Bzip2, LZ4, and Snappy [27].

### 2.3 Non-Hadoop Simultaneous Data Compression and Encryption

Hadoop’s framework was designed without any support for simultaneous data compression and encryption. In fact, simultaneous compression and encryption is a relatively new area of research. Its objective is to avoid separate operations for compression and encryption. Current state-of-the-art research in simultaneous data compression and encryption utilizing non-linear dynamic systems is now discussed.

From the 1990s, chaos theory in nonlinear dynamical systems had been applied in the development of low complexity cryptosystems. The intrinsic properties of non-linear dynamical systems such as ergodicity, mixing, sensitivity to the initial condition, control parameters, odd behaviors, etc., [28–30] are useful for the practical implementation of cryptosystems. These properties are directly linked to the confusion and diffusion properties of classical cryptosystems [31]. However, unlike chaos-based cryptography, which has widely accepted, the chaos-based simultaneous source coding and encryption is insufficiently studied [32–34]. Nevertheless, the realities of overcrowded networks combined with inadequate data storage capacity as well as growing network vulnerabilities, attacks, intrusions, big data challenges and various security threats have contributed to rising interests in investigating joint compression and encryption operations. Therefore, many chaos-based joint compression and encryption algorithms have been designed. Among them, low-dimensional chaotic maps are usually employed to protect the compressed data due to their high operating efficiency.

There are two different research tracks in the field of simultaneous compression and encryption. The first approach embeds encryption within compression algorithms while the second incorporates compression within cryptographic schemes. Both have limitations. Approaches based on Huffman coding—which uses tree mutation to increase the number of Huffman tables for simultaneous compression and encryption—have been presented in the literature [35–37]. The Huffman coder is readily used for encryption without complexity. Its encryption process introduces a secret key to control the Huffman tree and the same key is required to correctly decode encrypted data. Thus, the embedding of encryption features mainly attends the control of tree branch swapping by using the key. Wu and Kuo [35] presented an integrated compression and encryption approach that swapped Huffman tree branches, left and right, using the control key. Later, multiple code word issues were overcome by an improved approach that employed chaotically mutated Huffman trees [36]. This scheme also served to enlarge the main space and solve security issues [35]. The work of [36] was further improved by [37]. However, the length of the code word obtained by the statistical model remained unchanged and the resulting system was vulnerable to known-plaintext attack (KPA).

Research on embedding encryption into arithmetic coding has also been done, with notable examples [38–42]. A modified version of arithmetic coding was presented by [40] using key-based interval splitting to achieve simultaneous data compression and encryption. However, its vulnerability to known-plaintext attack was exposed by [43]. An improved approach was then presented by [39] who removed the interval constraint by splitting the AC interval, whereby two permutations were implemented for diffusion to enhance the security of the compression process. Also, chaotic systems were employed by [41,42] for key control that achieved simultaneous data compression and encryption. However, the chaotic system can only be used as a pseudorandom bitstream generator that incorporates key control. In another related work, a randomized arithmetic coding RAC algorithm [38] was proposed for the JPEG 2000 standard that inserted randomization protocols into the conventional arithmetic coding operation for purposes of encryption. However, both approaches by [43,44] demonstrated inferior output compared to the standard approach. These algorithms still posed serious security issues [41,43,44], as the relationship between chaos, encryption and compression had not been thoroughly investigated. Chaos systems were only used as pseudorandom bitstream generators that incorporated key controls.

#### 2.3.1 Source Coding using Generalized Luroth Series (GLS)

The relationship between source coding and chaos was studied by [32,34]. A non-linear chaotic dynamic system referred to as the Generalized Luroth Series (GLS) was proposed by [32] and proved to be Shannon optimal. For the purpose of source coding [32], the Tent map was defined as follows:

$$f(x)=\left\{ \begin{array}{cc} x/p & x\epsilon [0,p]\\ \left(1-x)/(1-p\right) & x\epsilon [p,1] \end{array}\right.$$ (1)

Where *p* is a probability between (0 and 1) and compression is attained by the inverse of the Tent map [32]:

$$I\{i\}=\left\{ \begin{array}{cc} p(I\{i-1\}) & if\;0\\ 1-p(I\{i-1\}) & if\;1 \end{array}\right.$$ (2)

Where *p* is probability and *I*{*i*} is the interval for the *i*th symbol. Initially, *I*{0} = [0,1]. Binary sequence encoding is performed by an iterative application of Eq (2) until the first symbol is encountered. At the end of the encoding process, the final interval [START, END] is obtained. Any value between the final interval, e.g. (START + END)/2, is converted into a binary compressed sequence. During decoding, the decoder requires this value to map through iterations of Eq (1) until the first symbol is encountered.

The GLS coding is conceptually simple, but its implementation is challenging due to the infinite precision of real numbers that result from the long products of real numbers. It requires splitting of the interval between (0 and 1) at every iteration of Eq (2). This division operation shrinks the range so fast it makes GLS coding unfeasible for encoding a larger sequence of 1000 bits. The experiments are conducted by implementing GLS coding over single-precision (32-bit) and double-precision (64-bit) IEEE 754 floating point formats to determine the maximum length of the binary sequence that can be encoded using GLS coding. The criteria is very simple, the maximum length of binary sequences that can be encoded should be greater than 32-bit or 64-bit in the case of single or double precision implementation, respectively.

**Fig 3:**
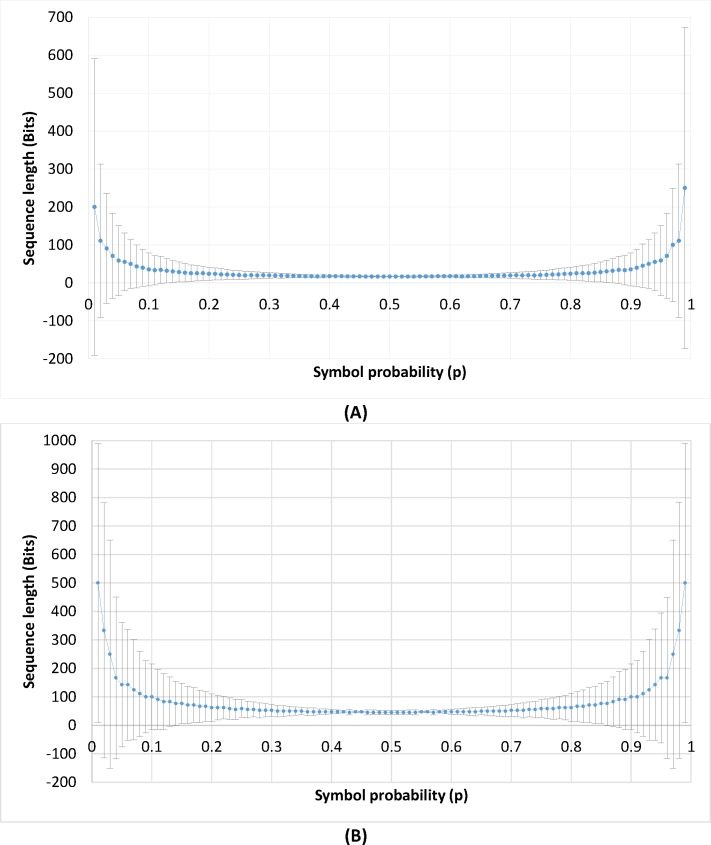
Experiment results to determine the maximum binary sequence length that can be encoded using GLS coding. (A) GLS coding results on a 32 bit floating point format. (B) GLS coding results on a 64 bit floating point format.

The 100 binary sequences are generated with a length of 1000 bits where each sequence has a different probability from 0 to 1. Results are shown in Fig 3 for computed means and standard deviations of all obtained binary sequence lengths at a specific probability. The maximum received bitstream length is relatively better for extreme probabilities that are *p* ∈ (0,0.1) *or* (0.9,1) and which remain greater than 32-bit and 100-bit in single and double precision implementation, respectively. However, maximum obtained bitstreams remain less than the desired lengths for both single and double precisions implementation for probabilities *p* ∈ (0.11,0.89). Thus, GLS Coding cannot be applied for compression purposes.

#### 2.3.2 Source Coding Using a Chaotic Symbolic Approach

A new theoretical concept for source coding based on chaotic modulation was presented by [34] for multiple symbols that demonstrated optimal entropy criteria for data compression. They introduced the probabilistic Bernoulli generator as a piecewise linear Markov map (PWLM) to map a sequence into an initial condition associated with the generator. In [45,46], this concept was implemented to achieve simultaneous source coding and encryption for a message sequence of *n* distinct symbols (*s*_1_,*s*_2_,……,*s*_*n*_) with probabilities (*p*_1_,*p*_2_,……,*p*_*n*_), respectively. Based on input data, a complex PWLM was constructed [34] as follows:

$$f(x)=\left\{ \begin{array}{cc} \left[\frac{x}{p_{1}}\right] & x\epsilon I_{1}\\ \left[\frac{x-p_{1}}{p_{2}}\right] & x\epsilon I_{2}\\ \begin{array}{c} \cdots \\ \left[\frac{\left(x-\sum_{i=1}^{n-1}p_{i}\right)}{p_{n}}\right] \end{array} & x\epsilon I_{n} \end{array}\right.$$ (3)

Where *i* = (1,2,3,………,*n*); *p*_*i*_ = probabilities; and intervals *I*_*i*_ are given as:

$$I_{1}=[0,p_{1}]$$ (4)

$$I_{i}=[\sum_{j=1}^{i-1}p_{j},\sum_{j=1}^{i}p_{j}],\;i=2,3,\ldots \ldots \ldots ,n$$ (5)

Intervals *I*_*i*_ associate with message sequence symbols *s*_*i*_ where *i* = 2,3,………,*n*.

Compression is achieved by reverse interval mapping [34] as follows:

$$I\{i\}=f_{p}^{-1}(I\{i-1\})=p_{n}(I\{i-1\})+\sum_{i=1}^{n-1}p_{i}$$ (6)

Where *I*{*i*} is the obtained interval of the *i*th symbol (*i* = 1,2,3………), and the initial interval is *I*{0} = [0,1]. To demonstrate the algorithm’s functionality, let *M* = *EFGH* be the sub message sequence to be encoded where the original message consists of 100 different symbols where each symbol has the same 0.01 probability. By this means, the first symbol has range of 0 to 0.01; the second from 0.01 to 0.02 and so on.

Message reading starts in reverse order from the last symbol (H) where the initial interval [0,1] is used to obtain the new interval, i.e. (0.07,0.08). The second last symbol is (*G*); hence, the interval (0.07,0.08) becomes (0.0607,0.0608). The third last symbol is (*F*) and the new interval (0.050607,0.050608) derives from (0.0607,0.0608). The message encoding process continues until the first symbol (*E*) is reached and the final interval is obtained (0.04050607,0.04050608). Any value within the final interval, e.g., (START + END)/2 = 0.040506075 can be stored as a compressed binary sequence. To perform decoding, the binary compressed sequence is converted to its original form. Furthermore, the obtained value is used as the initial value to iterate Eq (5) of PWLM and correctly determine the original message sequence, *M*.

**Fig 4:**
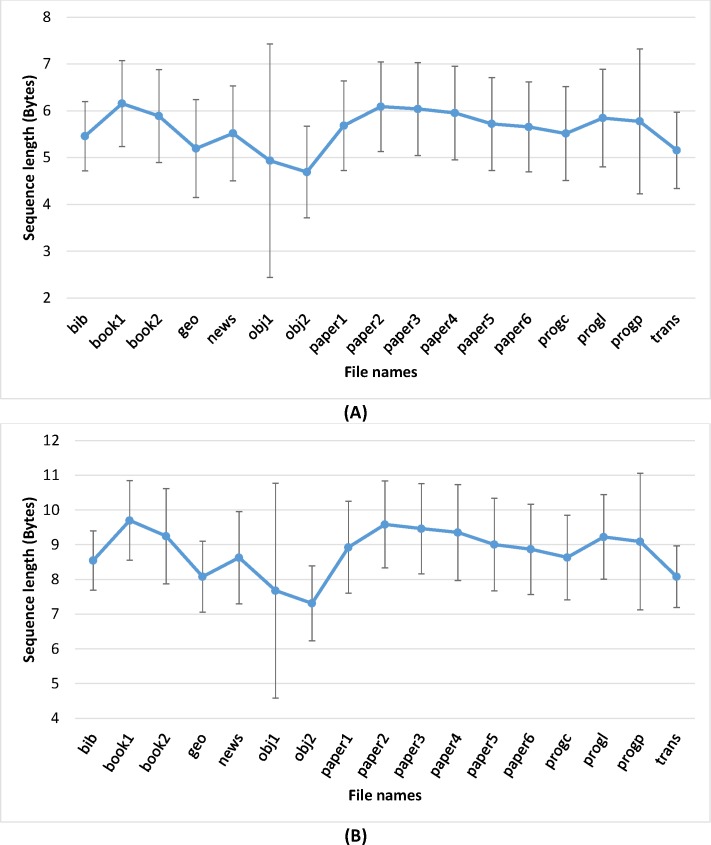
Experiment results to determine the maximum binary sequence length that can be encoded using PWLM coding. (A) PWLM coding results on a 32 bit floating-point format. (B) PWLM coding results on a 64 bit floating-point format.a 64 bit floating point format.

The implementation problem of infinite precision real numbers also exists in the PWLM source coding method. This requires a splitting of intervals between 0 & 1 for each iteration of Eq (6). This division operation shrinks the range so fast it makes PWLM coding unfeasible for the encoding of larger byte sequences. Experiments are conducted by implementing PWLM coding over single-precision (32-bit) and double-precision (64-bit) IEEE 754 floating point formats to determine the maximum length of byte sequences that can be encoded with PWLM coding. The criteria are very simple: the maximum length of byte sequences that can be encoded should be greater than 32-bits or 64-bits in the case of single or double precision implementation, respectively. Fig 4 shows mean and standard deviation results for selected files from norm Calgary Corpus files [47]. The maximum obtained byte sequence length is higher than 4-bytes and 8-bytes for single and double precision implementation, respectively, but the error bar indicates inconsistency. Experimental results confirmed it was possible to implement PWLM coding over 32-bit or 64-bit floating-point formats but in either case compression efficiency remained inadequate. Compression ratio results are reported in Section 4 for PWLM coding over a 64-bit floating-point format.

Both GLS and PWLM coding techniques are conceptually simple but their implementation is challenging due to the infinite precision of real numbers that result from the reverse interval mapping of real numbers. The obvious solution is to perform approximate computations and express these numbers as mantissa and exponents [48]. However, this leads to carry-over and positioning issues. Another solution, as introduced by the present work, is to add range coding, i.e., to bring all fractions to a common denominator.

## 3 Preliminaries

### 3.1 Skew Tent Map

The Skew Tent map (STM) has attracted substantial research focus in the past few years due to its high sensitivity to the initial condition and control parameter with good pseudo-randomness and ergodicity. It is a one-dimensional chaotic map that satisfies uniform distribution, invariant density and non-invertible transformation of the unit interval onto itself [49–51]. Eq (7) defines the iterative function of STM:

$$x_{n+1}=F_{\lambda }(x_{n})=\left\{ \begin{array}{c} \frac{x_{n}}{\lambda }\;if\;x_{n}\leq \lambda \\ \frac{{1-x}_{n}}{1-\lambda }\;if\;x_{n}\geq \lambda \end{array}\right.$$ (7)

**Fig 5:**
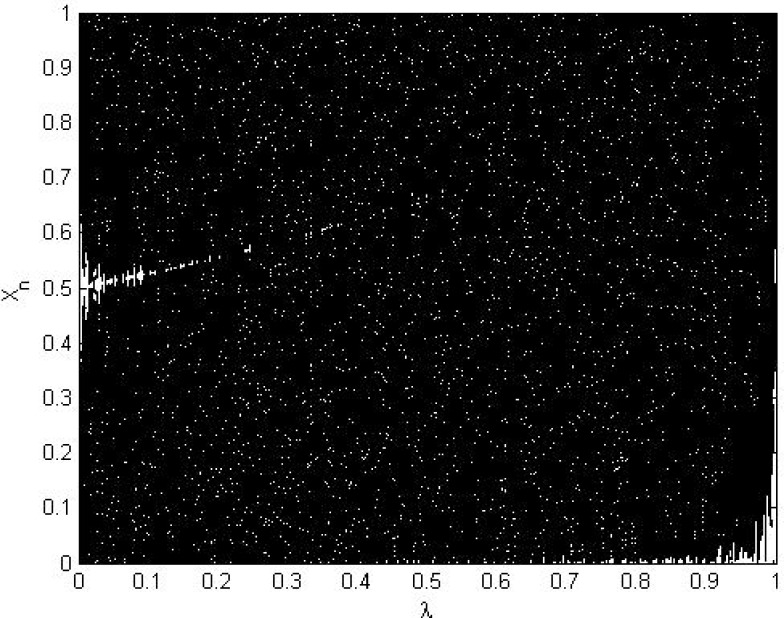
Bifurcation diagram of the STM.

**Fig 6:**
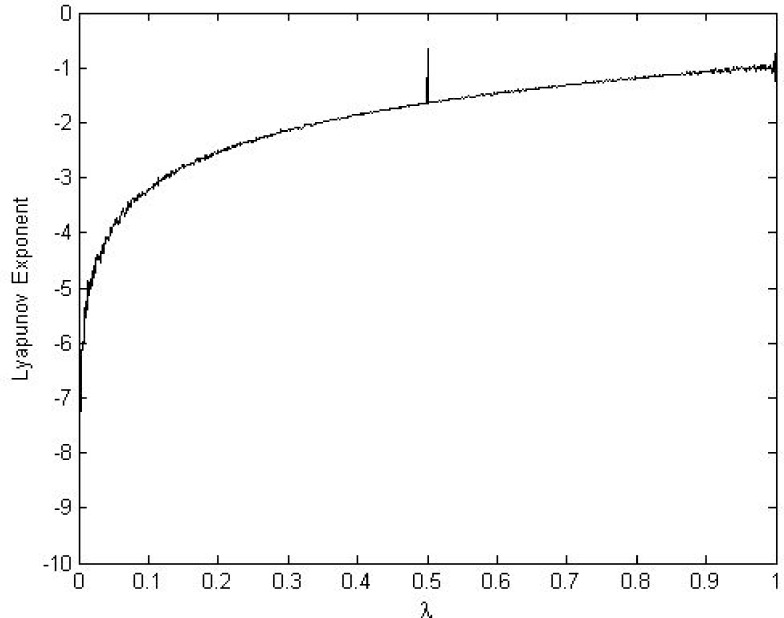
Lyapunov Exponent of the STM.

Where *λ* ∈ (0,1) is a control parameter *x*_*n*_ ∈ (0,1), and *x*_0_ represents the initial value at (*n* = 0). The orbits ${\left\{x_{n}\right\}}_{n=0}^{\infty }$ of STM are uniformly distributed over the interval [0, 1] and it has a positive Lyapunov exponent that validates its chaotic behavior [51]. The chaotic property of CTM is shown in bifurcation and the Lyapunov Exponent analysis in Figs 5 and 6, respectively. Both analyses results demonstrate that the transformation of the STM is continuous and piecewise linear and that its chaotic range is *λ* ∈ (0,1). However, work by [52,53] showed that STM is insufficiently random under finite precision. They then suggested a *λ* value from (0.25,0.49) or (0.51,0.75) to achieve sufficient randomness in STM output. Due to previously mentioned properties, the design proposed in the present paper utilizes the suggested value (*λ*) to generate a pseudorandom keystream for the chaotic masking operation.

### 3.2 The Chaotic Logistic Map

The chaotic Logistic map (CLM) is a well-known one-dimensional map that satisfies uniform distribution and invariant density. It demonstrates complex chaotic behavior and has strong cryptographic properties such as initial value sensitivity, erratic behavior and unpredictability, etc. [49,54,55]. Eq (8) defines CLM:

$$x_{n+1}=F_{\lambda }(x_{n})=\lambda x_{n}(1-x_{n})$$ (8)

**Fig 7:**
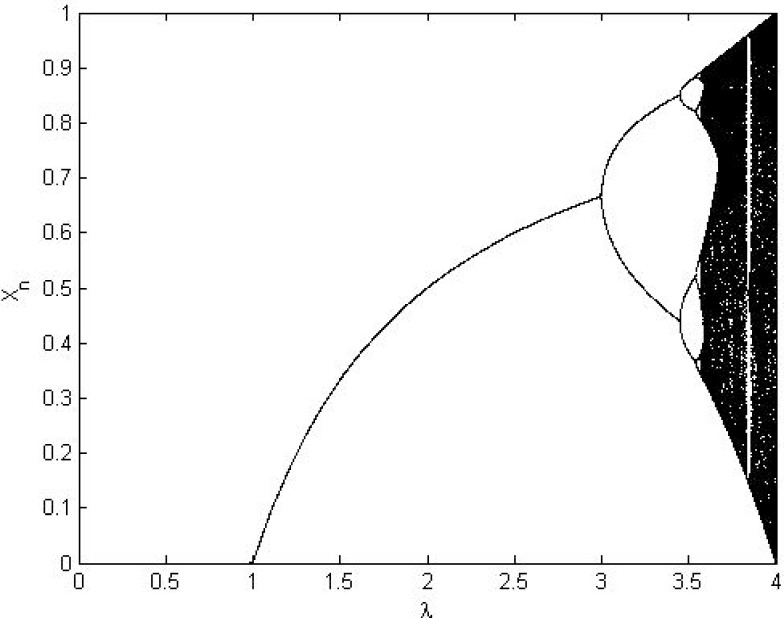
Bifurcation diagram of the CLM.

**Fig 8:**
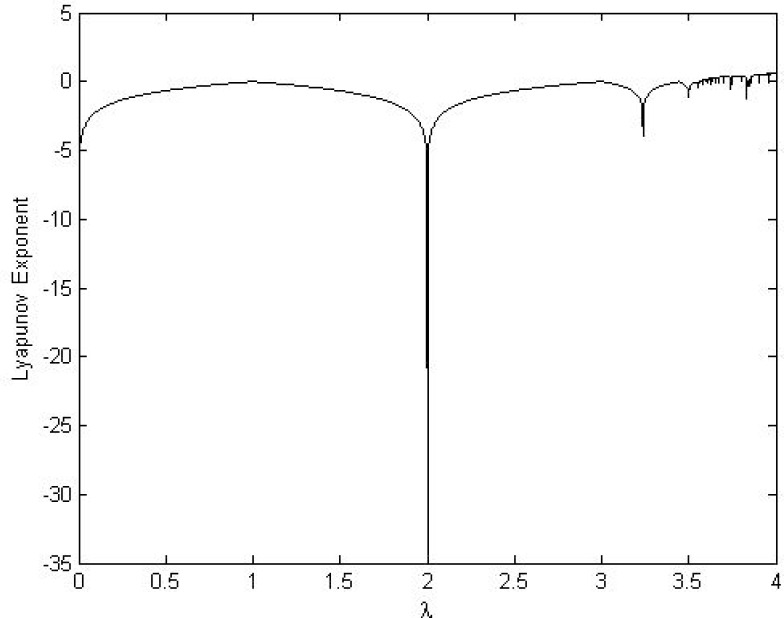
Lyapunov Exponent of CLM.

Where *x*_*n*_ ∈ (0,1); *n* = 0,1,2…..; *λ* ∈ (0,4); *x*_0_ is initial value when *n* = 0. Figs 7 and 8 show diagrams for bifurcation and the Lyapunov Exponent analysis, respectively. Both analyses show that the CLM exhibits chaotic behavior beyond *λ* = 3.57. However, certain isolated values of (*λ*) have non-chaotic behavior. The orbits ${\left\{x_{n}\right\}}_{n=0}^{\infty }$ of CLM are uniformly distributed over the interval [0, 1]. Based on these results, the design proposed in the present work utilizes the suggested value of (*λ*) to generate a pseudorandom keystream to the perform cyclic shift operation for the proposed source coding method without affecting its compression capabilities.

## 4 The Proposed Work

### 4.1 Source Coding Using Modified PWLM

The present work addresses the implementation of source coding using PWLM [34], which is a problem due to the infinite precision of real numbers consequent to the reverse interval mapping of real numbers. An improved source coding method is presented that increases the range from [0,1] to [0,*r*], where *r* is defined as *r* = *b*^*n*^ − 1, where *b* is base and *n* is the largest base-b number that the algorithm can conveniently handle. Performing interval re-scaling for each PWLM iteration into its last obtained interval can solve the second issue of reverse interval mapping. A detailed description now follows:

Let *n* be distinct symbols (*s*_1_,*s*_2_,……,*s*_*n*_) in a message sequence with probabilities (*p*_1_,*p*_2_,……,*p*_*n*_), respectively, where $\sum_{i=1}^{n-1}{rp}_{i}=r$ and *r* = *b*^*n*^ − 1; and where *b* is base and *n* is the largest number of base *b* that the algorithm can conveniently handle. Based on data, a PWLM is constructed as follows:

$$f(x)=\left\{ \begin{array}{cc} \left[\frac{x}{{rp}_{1}}\right]r & x\epsilon I_{1}\\ \left[\frac{x-{rp}_{1}}{{rp}_{2}}\right]r & x\epsilon I_{2}\\ \begin{array}{c} \cdots \\ \left[\frac{\left(x-\sum_{i=1}^{n-1}{rp}_{i}\right)}{rp_{n}}\right] \end{array}r & x\epsilon I_{n} \end{array}\right.$$ (9)

Where *i* = (1,2,3,………,*n*); *p*_*i*_ are probabilities; Intervals *I*_*i*_ associate with plaintext symbols *s*_*i*_ are given as:

$$I_{1}=[0,{rp}_{1}]$$ (10)

$$I_{i}=[\sum_{j=1}^{i-1}{rp}_{j},\sum_{j=1}^{i}rp_{j}],\;i=2,3,\ldots \ldots \ldots ,n$$ (11)

The compression is achieved by the forward interval mapping method as follows:

$$I\{i\}=f_{rp}^{-1}(I\{i-1\})=a_{i}+(b_{i}-a_{i})\times rp_{n}(I\{i-1\})+\sum_{i=1}^{n-1}rp_{i}$$ (12)

Where (*i* = 1,2,3 ………); *I*{*i*} is the obtained interval of the *i*th symbol; and where *a*_*i*_ and *b*_*i*_ are upper and upper values of the intervals, respectively. Here is where a major problem can occur while selecting the initial range, which must be sufficiently large regardless of how many symbols need encoding. Thus, the algorithm’s current range should be broad enough to allow division into a non-zero sub-range. As a solution for practical implementation, the encoder begins with a large range. For 32-bit implementation, the range calculation assumes *r* = *b*^*n*^ − 1, where *b* = 2 and *n* = 32, which means the starting range should be *r* = 2^32^ − 1.

To demonstrate the algorithm’s functionality and effective forward interval mapping, we encode the example taken from Section 2.3.2 using the proposed method with an initial range of *I*{0} = [0,1000000000]. For convenience, range *r* = *b*^*n*^ is calculated in the base 10 decimal system where *b* = 10 and *n* = 9. Let, *M* = *EFGH* be the sub-message sequence to be encoded where the original message consists of 100 different symbols. Thus, each symbol has a 10000000 probability. Thus, the first symbol ranges 0 to 10000000, second from 10000000 to 20000000 and so on. Message reading begins in forward order from the first symbol (E), where the initial interval [0,1000000000] obtains the new interval, i.e., (40000000,50000000). The second symbol is (*F*); hence, the interval (40000000,50000000) becomes (40500000,40600000). The third symbol is (*G*) and the new interval (40506000,40507000) derives from (40500000,40600000). The message encoding process continues until the last symbol (*H*) is reached and the final interval is obtained (40506070,40506080). Any value within the final interval; e.g., (START + END)/2 = 40506075 and can be stored as a compressed binary sequence. For decoding, the binary compressed sequence is converted to its original form. Furthermore, the obtained value is used as the initial value to iterate Eq (9) of PWLM and correctly determine the original message sequence, *M*.

Of interest, the final interval obtained from PWLM coding is the same as the proposed method but without any fractional component. Secondly, it becomes evident that after encoding a number of symbols the leftmost digits of the range do not change. Thus, the encoder repeatedly checks the leftmost digits within the range for adjustments, after which scale numbers are changed on the right as figures on the left are removed. The decoder applies the same approach and remembers current values retrieved from Eq (9) of PWLM. The decoder simply removes the value and inserts a new value retrieved from Eq (9) of PWLM. Hence, and based on experimental studies, the proposed method confirms the correspondence and generalization of arithmetic coding. The source coding method presented in [34] is similar to the proposed method but has an implementation issue of infinite precision for real numbers that result from the reverse interval mapping of real numbers. The method proposed herein solves the implementation issue by introducing forward interval mapping and the removal of fractional components generated by the long products of real numbers. Moreover, the proposed method incorporates a stealth key that performs a cyclic shift in PWLM without compromising compression capabilities. In addition, the proposed approach implements a masking pseudorandom keystream that enhances encryption quality.

### 4.2 Pseudorandom Keystream Generator

The Logistic and Tent maps were selected as underlying chaotic maps for pseudorandom keystream generators to introduce confusion and diffusion in the proposed method. Both Tent and Logistic maps are iterative (starts from an initial condition) and obtain *x*_*n*_ values from Eqs (7) and (8), respectively. Obtained *x*_*n*_ values are converted into binary sequences by defining a threshold or *t* value. Here, the fair coin model is adopted to generate binary sequences from both chaotic maps. Based on the fair coin model, the probability of each symbol is 0.5, thus the threshold value is set as *t* = 0.5 and defined by Eq (13):

$$B_{n}=\left\{ \begin{array}{cc} 0 & 0\leq x_{n}<t\\ 1 & t\leq x_{n}\leq 1 \end{array}\right.$$ (13)

**Fig 9:**
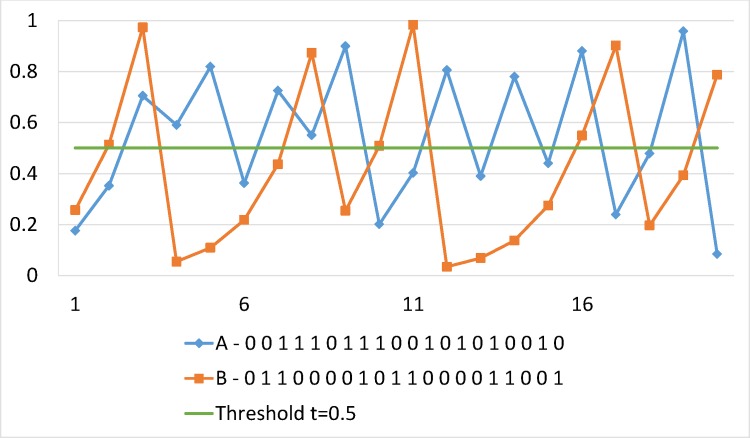
Illustration of two binary sequences using Tent Map. Trajectories indicated by *x*_0_ = 0.1758 and *x*_0_ = 0.2563, where *λ* = 0.4995.

**Fig 10:**
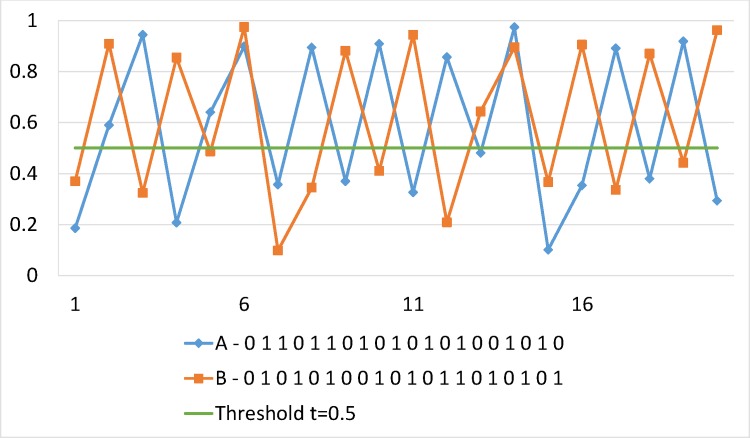
Illustration of two binary sequences using Logistic Map. Trajectories indicated by *x*_0_ = 0.1856 and *x*_0_ = 0.3695, where *λ* = 3.99.

Binary sequences are obtained from real values (*x*_*n*_) of both Logistic and Tent maps by comparison with thresholds (See: Figs 9 and 10). Finally, a statistical analysis is performed on generated binary sequences using the stringent NIST statistical test suite for randomness to detect specific characteristics that mark truly random sequences. The latter allows them as excellent choices for pseudorandom keystream generation. Analyses results are reported in Section 5.2.4.

### 4.3 Simultaneous Data Compression and Encryption

**Fig 11:**
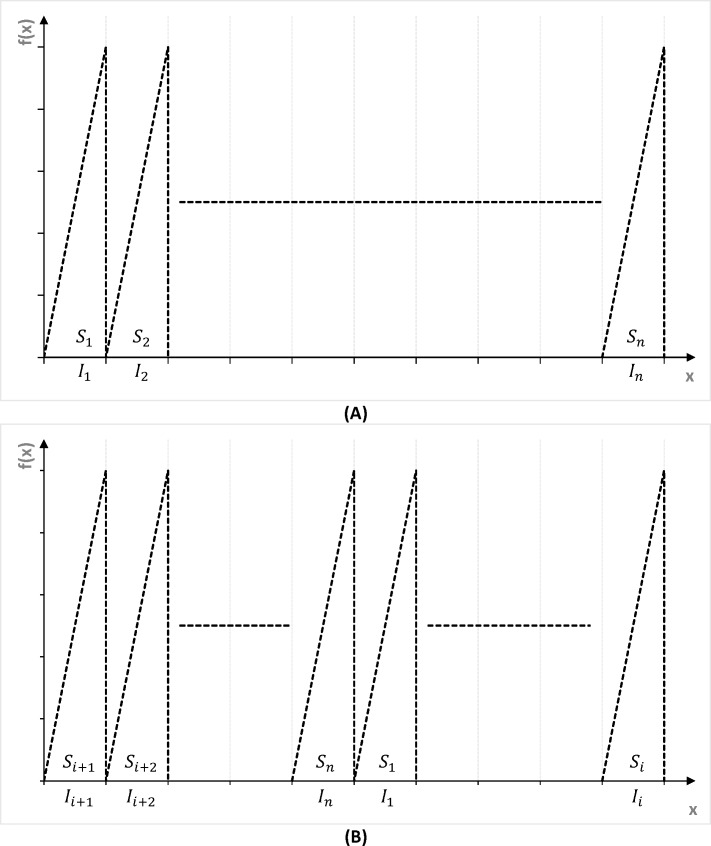
Trajectory of PWLM. (A) Trajectory without cyclic-shift when *KC*_*i*_ = 0. (B) Trajectory with cyclic-shift when *KC*_*i*_ = *i*.

Data encoding is performed using Eq (12) of PWLM and by introducing two secret keys (*K*1,*K*2) that control the PWLM mode as it performs the masking operation where each key is a combination of *x*_0_ and *λ*. This process generates keystreams by using the pseudorandom keystream generator. The encoder begins with a larger number range *r* = 2^32^ − 1 (as mentioned in section 4.1) and is further adjusted during encoding or decoding. Data encoding steps are defined as follows:

1. Encoding begins with reading (scanning) bytes of plaintext (*P*) to determine *n* number of distinct symbols (*s*_1_,*s*_2_,……,*s*_*n*_) and corresponding probabilities (*p*_1_,*p*_2_,……,*p*_*n*_), respectively. The plaintext is then divided into *N* blocks of 1024 bytes where the last block can contain *B* ≤ 1024 bytes. The numbers of blocks, distinct symbols and corresponding probabilities are then stored in a compressed file as header data.
2. Importantly, one must select an initial range that is sufficiently broad to encompass all possible symbols that require encoding. Hence, this range should be sizeable enough for division into a non-zero sub-range.
3. Next, the first secret key (*K*1) generates a keystream by using the pseudorandom keystream generator (size *N* × 8 bits) according to *N* number of blocks in plaintext, *P*. The keystream is then divided into *N* bytes that generate cyclic shift keys (*KC*_1_,*KC*_2_,……,*KC*_*N*_) or (*KC*_*i*_) where (*i* = 1,2,……,*N*). The key (*KC*_*i*_) is then used to control the PWLM mode. The (*KC*_*i*_) is taken as a secret number (*KC*_*i*_ *ϵ* 0,1,………,255) to cyclic-shift the position. Fig 11 presents a simple example of PWLM mode control.
4. Based on plaintext statistical analysis, the first block is then encoded for each symbol via a forward interval function beginning with the first symbol, whereby compression and encryption are simultaneously completed until reaching the last symbol. The process adjusts the range by repeatedly checking the leftmost digits of the range for adjustments, after which scale numbers are changed on the right as figures on the left are removed. After the block’s 1024 symbols are processed, the final [START, END] interval is determined. Values with binary representation within this final range are selected to encode data; e.g., (END+START)/2. Also, an “End” character is placed at the end of the processed block and then scanned to determine *n* number of distinct blocks. Here, a special character (011111111) comprising one “0” and eight “1s” is added at the end of the binary sequence. If a sequence has seven consecutive “1s”, a “0” is then added after the seventh “1”. Subsequently, remaining blocks are encoded in the same manner until all blocks are completed.
5. Finally, the second key (*K*2) generates a pseudorandom keystream and masks the completed compressed file that now comprises header data and compressed blocks. The masking operation is performed by a simple XOR operation, which augments the overall randomness of encoded data and further secures header data.

### 4.4 Simultaneous Data Decompression and Decryption

Decoding reverses the order of the encoding process. The initial decoding range begins at *r* = 2^32^ − 1 and is further adjusted as described in the encoding section. The process proceeds as follows:

1. Initially, the second key (*K*2) generates a pseudorandom keystream and unmasks the completely compressed file. Header data is then read to identify distinct plaintext symbols (*s*_1_,*s*_2_,……,*s*_*n*_) as well as corresponding probabilities (*p*_0_,*p*_1_,……*p*_255_) and *N* number of blocks.
2. Next, the secret key (*K*1) generates a keystream using the pseudorandom keystream generator of *N* × 8 bits according to *N* number of plaintext (*P*) blocks. This keystream is then divided into *N* bytes to generate cyclic shift keys (*KC*_1_,*KC*_2_,……,*KC*_*N*_ or *KC*_*i*_), where (*i* = 1,2,……,*N*).

The decoder now reads all compressed data and scans all binary data for code words. When it encounters the first “0111111111” character, code words for the first block are retrieved and the next sequence with “0” (after seven consecutive “1”s) is removed. Based on the first block’s statistical properties, it is now simultaneously decompressed and decoded according to the forward iteration of Eq (9). This rendering follows the cyclic shift key (*KC*_*i*_), by which the corresponding block and each symbol are adjusted for range. All remaining blocks are similarly decoded until the plaintext (*P*) is fully recovered.

## 5 Experiment Analysis

### 5.1 Comparison with Source Coding using PWLM

Tests for source coding between PWLM and the proposed algorithm utilized a Pentium-IV 2.4 MHz PC (Windows 7 and 3 GB RAM) without the Hadoop framework to determine the effectiveness of the proposed algorithm by bringing all fractions to a common denominator. Both algorithms employed Java and were benchmarked with Calgary Corpus files [47]. Measured performance parameters were ‘compression ratio’ and ‘execution time.'

#### 5.1.1 Compression Ratio

Table 1 shows compression ratio results for selected files from the standard Calgary Corpus. Eq (14) was used to calculate all compression ratios:

$$Ratio=1-\frac{ciphertext\;length}{plaintext\;length}\times 100\%$$ (14)

**Table 1:** Comparison of compression ratios.

File	Size KB	Proposed Algorithm %	PWLM Coding %
**book1**	768771	40.84	13.19
**book2**	610856	37.78	8.93
**paper2**	82199	39.82	10.27
**paper3**	46526	38.88	7.66
**progl**	71646	37.36	6.60
**progp**	49379	36.10	4.14
**Avg. Compression ratio**		38.46	8.46

Computed average compression ratios were 38.46 for the proposed algorithm vs. 8.46 for source coding with PWLM. Experimental results demonstrated that the enhancement of PWLM source coding improved compression capabilities.

#### 5.1.2 Algorithm Speed

Table 2 summarizes both encryption and decryption periods for both algorithms. Averaged execution periods clearly indicated a performance edge for the proposed algorithm, which took less time for both encryption and decryption processes.

**Table 2:** Comparison of encryption and decryption periods.

	Encryption time (seconds)		Decryption time (seconds)	
Filename	Proposed Algorithm	PWLM Coding	Proposed Algorithm	PWLM Coding
**book1**	312	441	400	1844
**book2**	130	340	320	1440
**paper2**	20	40	40	210
**paper3**	10	20	30	110
**progl**	10	30	40	170
**progp**	10	30	20	122
**Avg. Time**	82	150.17	141	649.33

#### 5.1.3 Results and Discussion

This section presents our comparison of results between Source Coding using PWLM and those of the proposed algorithm. Both algorithms were successfully implemented and thoroughly tested using selected Calgary Corpus files [47]. Experimental results validated an acceptable compression ratio for the proposed algorithm. Moreover, the enhanced version addressed the implementation issue of infinite precision real numbers by eliminating fractional components generated by the long products of real numbers in Source Coding using PWLM; thus representing an improvement that increased both speed and resource efficiency.

### 5.2 Security Analysis

#### 5.2.1 Key Space Analysis

The proposed algorithm utilizes two chaotic maps to generate pseudorandom keystreams. Each chaotic map requires two input parameters, an initial value (*x*_0_) and a control parameter (*λ*) to perform required operations. If both chaotic maps are realized in a finite precision system based on the double precision of floating point representation, then the secret key size is ~212 bits considering only the mantissa where the size of each input parameter is 53 bits. Thus, the size of the given secret key is large enough to resist brute-force attacks and fulfill cryptographic requirements.

#### 5.2.2 Key Sensitivity and Plaintext Sensitivity

A cryptographic algorithm should be highly sensitive to key and plaintext so that the slightest change in key or plaintext is reflected in its output. The changing of (*x*_0_ = 0.30896) to $\left(x_{0}^{\prime }=0.30897\right)$ assessed the secret key’s (*K*1) sensitivity. Trials were performed with Calgary Corpus files [47] where ciphertext files obtained from the same plaintext were compared, bit-by-bit, using two different keys as described above. Table 3 provides a comparison of results for key sensitivity trials. Similarly, plaintext sensitivity was evaluated by randomly toggling one bit while performing encryption with the same key. Finally, ciphertext files were compared bit-by-bit (See: Table 4). Moreover, bit-change-percentages for key and plaintext sensitivity were very close to the ideal value (50%); thus demonstrating that the proposed algorithm is highly sensitive to both key and plaintext as well as secure from cryptanalysis attacks.

**Table 3:** Key sensitivity analysis.

Filename	beginning	middle	end	complete
**book1**	49.415	49.413	49.411	49.413
**book2**	49.413	49.413	49.412	49.413
**paper2**	49.414	49.412	49.412	49.413
**paper3**	49.574	49.412	49.410	49.415
**progl**	49.418	49.412	49.411	49.414
**progp**	49.415	49.414	49.411	49.413

**Table 4:** Plaintext sensitivity analysis.

Filename	beginning	middle	end	Percentage
**book1**	49.137	49.134	49.066	49.112
**book2**	49.030	51.190	49.020	49.013
**paper2**	51.101	51.116	51.217	51.128
**paper3**	50.165	51.208	51.184	50.986
**progl**	49.056	50.487	49.004	51.149
**progp**	49.139	50.922	50.928	50.996

#### 5.2.3 Randomness Analysis of the Proposed Algorithm

The NIST SP800-22 [56] and DIEHARD [57] statistical test suites are the two most popular tools for randomness analysis. Their purpose is to rigorously analyze the binary sequence or ciphertext for randomness or non-randomness statistics. This study employed the NIST (National Institute of Standards and Technology) statistic test suite to evaluate the proposed algorithm’s security level. This suite comprises sixteen statistical tests; each test produces probability (*p*) values between zero and one to provide randomness statistics for the binary sequence or ciphertext. This probability value is used to determine acceptance or rejection by defining the level of significance (*α*). For example, when *p* < *α*, the binary sequence or ciphertext is non-random, if otherwise, the binary sequence or ciphertext is considered random. Here, the significance level (*α*) is adjusted to 0.01 to ensure 99% confidence for the randomness of the binary sequence or ciphertext generated by the proposed algorithm. Table 5 lists all computed *p* values for all tests. The proposed algorithm successfully passed all NIST tests and proved secure with a 99% confidence level.

**Table 5:** NIST Randomness test results.

Statistical test	Parameter	p-value	Result
**Frequency**		0.4399	Pass
**Block frequency**	M = 128	0.5486	Pass
**Runs**		0.7328	Pass
**Long runs of one’s**		0.2514	Pass
**Binary Matrix Rank**		0.7039	Pass
**Spectral DFT**		0.9076	Pass
**No overlapping templates**	M = 73023, N = 8, M = 9	0.0318	Pass
**Overlapping templates**	M = 9, M = 1032, N = 566	0.6905	Pass
**Universal**		0.6772	Pass
**Linear complexity**	M = 500, N = 1168	0.1248	Pass
**Serial**	m = 8, p_value1	0.6527	Pass
	m = 8, p_value1	0.1478	Pass
**Approximate entropy**	m = 10	0.9800	Pass
**Cumulative sums**	Forward	0.611	Pass
	Reverse	0.7767	Pass
**Random excursions**	x = -1	0.2855	Pass
**Random excursions variant**	x = -1	0.2142	Pass

#### 5.2.4 Randomness Analysis of Chaotic Pseudorandom Generators

The randomness analysis of both chaotic pseudorandom keystream generators was performed by generating 300 binary sequences of length *L* = 1,000,000 bits from each generator to evaluate required cryptographic properties of randomness for generated binary sequences. The control parameter (*λ*) values were taken randomly between *λ* ∈ (0.25,0.75) for the Tent map and *λ* ∈ (3.6,4) for the Logistic map. The initial values (*x*_0_) were taken randomly between *x*_0_ ∈ (0,1) for both generators. The significance level was adjusted to α = 0.01 to ensure 99% confidence for the randomness of pseudorandom binary sequences generated by Logistic and Tent maps. The range of the passing percentage for each test was determined using Eq (15):

$$P\pm 3\sqrt{P(1-P)/N}$$ (15)

Where *P* = 1 − α and *N* is the number of binary sequences. In our case, α = 0.01, *P* = 0.99 and *N* = 300; thus, the acceptable percentage range is between [97.28, 100]. If any test produced less than 97.28%, then binary sequences were considered non-random. Table 6 presents percentages for each test of pseudorandom binary sequences generators. All pseudorandom binary sequences were generated by using Logistic and Tent maps and successfully passed all NIST SP800-22 tests, as percentages remained between [97.28, 100]. Further, statistical analysis revealed that all generated binary sequences were independent and identically distributed random sequences with 99% confidence levels.

**Table 6:** Results of randomness analysis.

		Logistic map generator		Tent map generator	
Statistical test	Parameter	Passing %	Decision	Passing %	Decision
**Frequency**		99.67	Pass	99	Pass
**Block frequency**	M = 128	98.67	Pass	99.67	Pass
**Runs**		99.99	Pass	97.33	Pass
**Long runs of one’s**		99.33	Pass	99	Pass
**Binary Matrix Rank**		99.33	Pass	98.67	Pass
**Spectral DFT**		97.33	Pass	99	Pass
**No overlapping templates**	M = 73023, N = 8, M = 9	99.17	Pass	98.14	Pass
**Overlapping templates**	M = 9, M = 1032, N = 566	98.67	Pass	99	Pass
**Universal**		98.33	Pass	98.33	Pass
**Linear complexity**	M = 500, N = 1168	98.33	Pass	99.67	Pass
**Serial**	m = 8, p_value1	99.33	Pass	90.83	Pass
**Approximate entropy**	m = 10	99	Pass	98.33	Pass
**Cumulative sums**	Forward	100	Pass	99.33	Pass
**Random excursions**	x = -1	99.09	Pass	98.69	Pass
**Random excursions variant**	x = -1	98.85	Pass	99.11	Pass

### 5.3 Experimental Analysis in Hadoop

**Fig 12:**
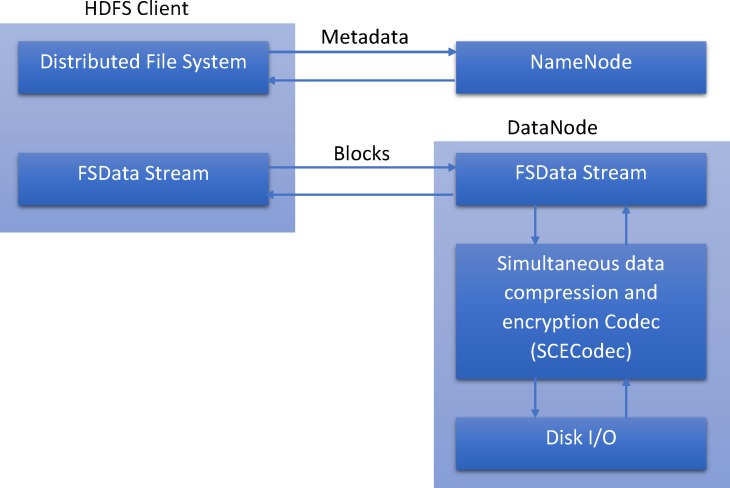
Block diagram describing the integration of the proposed algorithm in HDFS to read and write data.

The proposed algorithm was integrated into Hadoop using Java’s CompressionCodec to demonstrate the application of simultaneous data compression and encryption for Big Data. The new SCECodec (Simultaneous data compression and encryption codec) class was introduced to the codec library of Hadoop’s source code to perform simultaneous data compression and encryption. Fig 12 shows a block diagram of the proposed algorithm’s integration in HDFS to read and write data. A benchmarking suite was created to simulate read/write operations in HDFS to determine the compression ratio as well as performance metrics and security levels for the proposed algorithm’s handling of simultaneous data compression and encryption. A second objective was to identify whether or not MR jobs recognized all formats in HDFS by testing various file formats; e.g., text and compressed data with the proposed algorithm with Bzip2, Gzip and Lz4 algorithms. Experiments were performed with metocean data, which refers to forecasting and recording meteorological and oceanographic conditions at a specified location. This data format typically comprises well-defined parameters (wind speed, wind direction, atmospheric pressure, air temperature, waves, ocean currents, levels of water salinity, water temperature, sea level, etc.) [58]. Metocean data banks are vast, complex and continuous and rapidly grow; hence, their data storage requirements and transmission volumes are serious considerations.

#### 5.3.1 Compression Settings and Results

**Fig 13:**
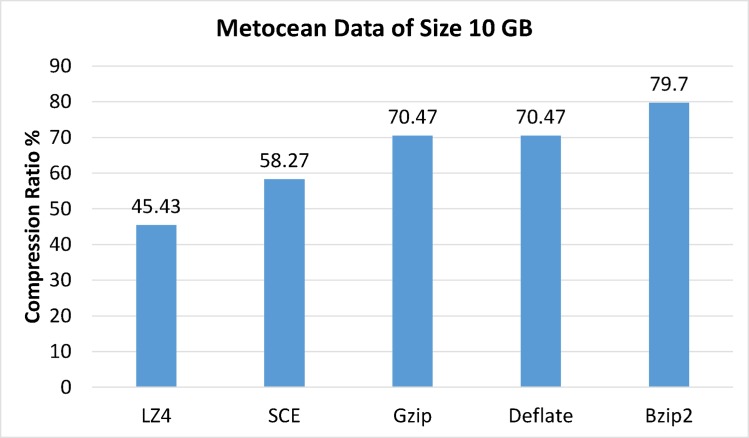
Compression ratio results.

Experiments were performed with 10GB of metocean data to evaluate the proposed algorithm’s compression capabilities compared with Hadoop’s Bzip2, Gzip, LZ4 and Deflate algorithms. The compression ratio was calculated using Eq (10). The same source files were used to run all trials without customizing either algorithms or default parameters while using default file extensions for the compressed files. Fig 13 shows experimental results and compression ratios for each codec. Results clearly demonstrated that all compressed file formats were recognized and accurately processed by HDFS without code changes.

#### 5.3.2 Processing Time Analysis

**Fig 14:**
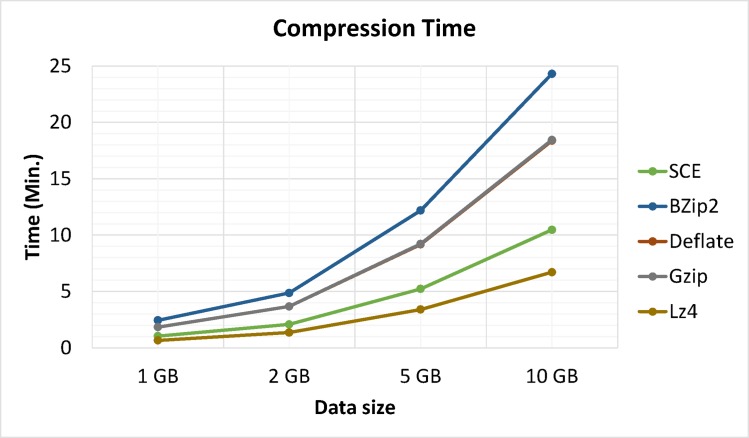
Processing times for compression.

**Fig 15:**
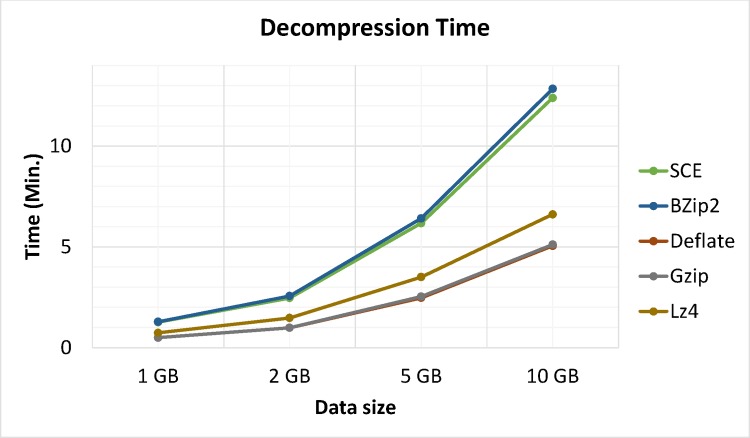
Processing times for decompression.

Apart from compression analysis, another important issue is processing time. Moreover, compression algorithms were optimized for either speed or compression but not both. Figs 14 and 15 show a comparison of compression and decompression processing times between the proposed algorithm and Bzip2, Gzip, LZ4 and Deflate algorithms on Hadoop for trials running metocean data sizes of 1, 2, 5 and 10 GB, respectively. Results showed that LZ4 and the proposed algorithm were the speediest. Comparatively speaking, Gzip, Deflate, and Bzip2 had better compression ratios. However, the latter algorithms cannot simultaneously compress and encrypt data.

**Fig 16:**
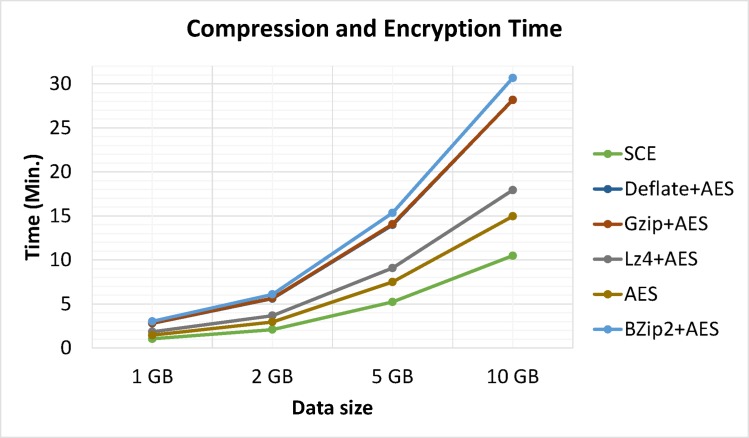
Processing times: compression and encryption.

**Fig 17:**
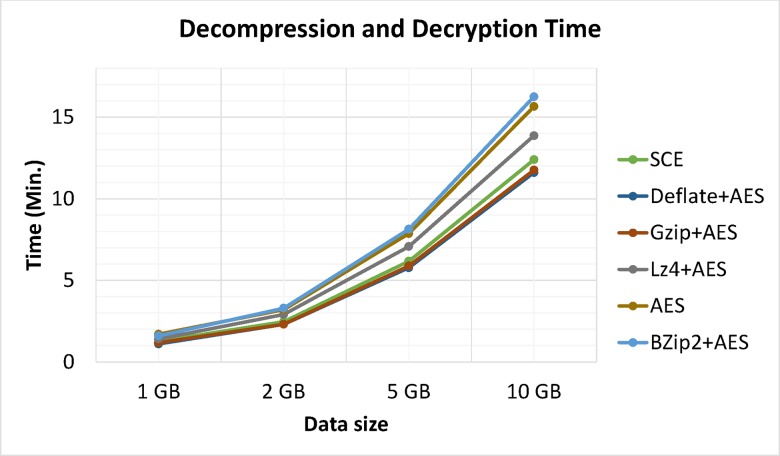
Processing times: decompression and decryption.

Test data was also compressed and encrypted by Bzip2, Gzip, LZ4 and Deflate algorithms with AES. Figs 16 and 17 show experimental results for different data sizes (1, 2, 5 and 10 GB). These results verified that the proposed algorithm was the fastest program compared to AES, Bzip2+AES, Gzip+AES, LZ4+AES, and Deflate+AES, and took less time to perform simultaneous data compression and encryption compared to two separate operations.

#### 5.3.3 Results and Discussion

**Fig 18:**
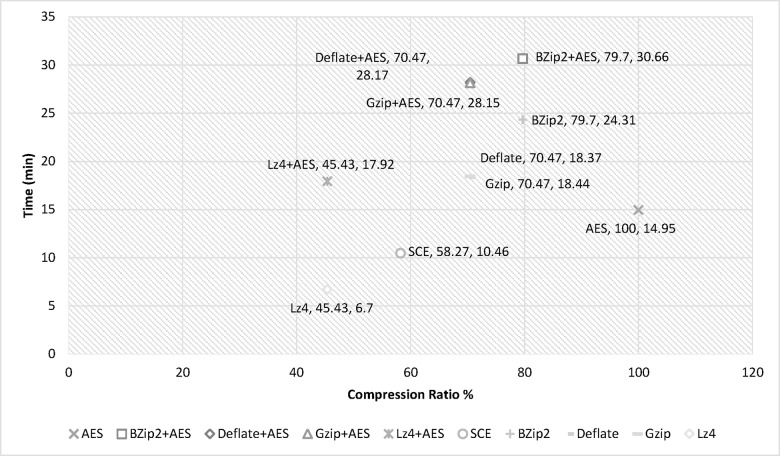
Space-Time tradeoff comparison.

Experimental results showed that LZ4 and the proposed algorithm had the fastest compression speeds, with Gzip in third place. Deflate, and Bzip2 had the slowest processing times for both compression and decompression but showed better compression ratios (See: Fig 18); thus showing a space-time tradeoff. It is important to note that Bzip2, Gzip, LZ4 and Deflate are limited to compression and are not considered secure, as they do not support simultaneous data compression and encryption. Furthermore, test data was compressed and encrypted by Bzip2, Gzip, LZ4 and Deflate algorithms followed by AES. Results clearly identified the proposed algorithm was superior, as it used less time while also providing adequate security and compression. Performing separate operations (compression and encryption) in Hadoop requires significant volumes of data piped between both operations. Hence, combining data compression with encryption as a single procedure reduces required data space and I/O network resource consumption during the process while also realizing data encryption.

## Conclusion

This work addressed the problem of implementing source coding with Tent Map and the Piece Wise Linear Markov map (PWLM), which require infinite precision real numbers consequent to long products of real numbers. A chaos-based simultaneous compression and encryption scheme for Hadoop was proposed to solve the implementation issue by removing fractional parts generated by long products of real numbers. The enhancement subsequently increased the algorithm’s speed and efficiency. In addition, a chaotic pseudorandom keystream generator controlled PWLM mode without compromising its compression capability. This approach incorporates a secret key by performing a cyclic shift operation in PWLM. Furthermore, introducing a masking pseudorandom keystream also enhanced encryption quality and the proposed algorithm also fit well within Hadoop’s framework to provide robust encryption security and compression while storing data. Experimental results established that the performance of separate operations for compression and encryption in Hadoop requires the piping of significant volumes of data between both processes, which degrades overall performance of the cluster. The proposed algorithm decisively achieved simultaneous data compression and encryption; thus proving useful during MapReduce jobs by reducing required data space and I/O cum network resource consumption. Our security analysis also revealed that the proposed algorithm is highly sensitive to both key and plaintext and that generated ciphertexts successfully passed all NIST SP800-22 assays; thus proving it is random with a 99% confidence interval.
