# Correction: Chaos-Based Simultaneous Compression and Encryption for Hadoop

**DOI:** 10.1371/journal.pone.0195420

**Published:** 2018-03-29

**Authors:** Muhammad Usama, Nordin Zakaria

There are typographical errors in Eqs (6), (7) and (12). Please view the corrected equations below.

$$I\left\{i\right\}=f_{p}^{-1}\left(I\left\{i-1\right\}\right)=p_{i}\left(I\left\{i-1\right\}\right)+\sum_{j=1}^{i-1}p_{j}$$ (6)

$$x_{n+1}=F_{\lambda }\left(x_{n}\right)=\{\begin{array}{c} \frac{x_{n}}{\lambda }\;ifx_{n}<\lambda \\ \frac{1-x_{n}}{1-\lambda }\;ifx_{n}\geq \lambda \end{array}$$ (7)

$$I\left\{i\right\}=f_{rp}^{-1}\left(I\left\{i-1\right\}\right)=a_{i-1}+\left(b_{i-1}-a_{i-1}\right)\times \left(\sum_{j=1}^{i-1}p_{j},\sum_{j=1}^{i}p_{j}\right)$$ (12)
